# Cyclodextrin Formulation of the Marine Natural Product Pseudopterosin A Uncovers Optimal Pharmacodynamics in Proliferation Studies of Human Umbilical Vein Endothelial Cells 

**DOI:** 10.3390/md11093258

**Published:** 2013-08-26

**Authors:** Daniel R. Day, Suraya Jabaiah, Robert S. Jacobs, R. Daniel Little

**Affiliations:** 1 Department of Ecology, Evolution, and Marine Biology, University of California, Santa Barbara, CA 93106, USA; E-Mails: daniel.richard.day@gmail.com (D.R.D.); rsjacobs@chem.ucsb.edu (R.S.J.); 2 Department of Molecular, Cellular, and Developmental Biology, University of California, Santa Barbara, CA 93106, USA; E-Mail: sjabaiah@ucsd.edu; 3 Department of Chemistry and Biochemistry, University of California, Santa Barbara, CA 93106, USA

**Keywords:** pseudopterosins, log P, human umbilical vein endothelial cells (HUVEC), proliferation, hydroxypropyl-beta-cyclodextrin (HPβCD)

## Abstract

Pseudopterosin A (PsA) treatment of growth factor depleted human umbilical vein endothelial cell (HUVEC) cultures formulated in hydroxypropyl-β-cyclodextrin (HPβCD) for 42 h unexpectedly produced a 25% increase in cell proliferation (EC_50_ = 1.34 × 10^−8^ M). Analysis of dose response curves revealed pseudo-first order saturation kinetics, and the uncoupling of cytotoxicity from cell proliferation, thereby resulting in a widening of the therapeutic index. The formulation of PsA into HPβCD produced a 200-fold increase in potency over a DMSO formulation; we propose this could result from a constrained presentation of PsA to the receptor, which would limit non-specific binding. These results support the hypothesis that the non-specific receptor binding of PsA when formulated in DMSO has ostensibly masked prior estimates of specific activity, potency, and mechanism. Collectively, these results suggest that the formulation of PsA and compounds of similar chemical properties in HPβCD could result in significant pharmacological findings that may otherwise be obscured when using solvents such as DMSO.

## 1. Introduction

**Figure 1:**
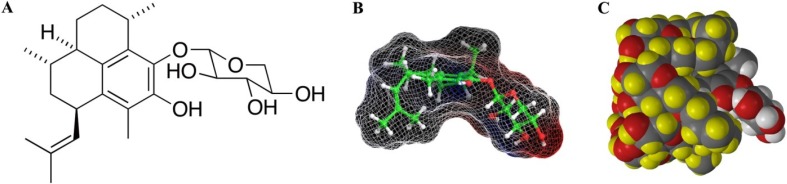
Chemical Structures. (**A**) Pseudopterosin A (PsA), (**B**) Molecular modeling of PsA, caged contour using H_2_O as a probe, (**C**) The result of molecular docking simulations using minimum energy conformations of hydroxypropyl-β-cyclodextrin with PsA (HPβCD-PsA) to form an inclusion complex (molecular mechanics calculations were performed using the Spartan 08 software package from Wavefunction, Inc.). HPβCD and PsA appear as space filling representations. The sugar unit of PsA protrudes from the cavity (note the white and red atoms).

Pseudopterosins represent a novel class of diterpene glycoside secondary metabolites (Figure 1) that are present in abundance in the marine gorgonian, *Pseudopterogorgia elisabethae*. Several naturally occurring pseudopterosin analogs have shown anti-inflammatory [1] and analgesic [2] activities. They have been shown to be unique topical and systemic anti-inflammatory agents that operate during the early stages of the inflammatory response by blocking phorbol 12*-*myristate-13*-*acetate (PMA) induced neutrophil infiltration and degranulation in the acute inflammatory response [2]. Initial *in vitro* studies indicated that PsA inhibited calcium ionophore induced degranulation and infiltration of human neutrophils [3]. Recent radio ligand binding studies with PsA, prepared in a DMSO formulation, resulted in micromolar binding affinities to isolated human adenosine A_1_, A_2A_, A_2B_, and A_3_ receptor subtypes with a high amount of non-specific binding [4].

A semi-synthetic analog, PsA methyl ether, has shown topical efficacy in pre-clinical models of wound healing [5]. Since cell proliferation/angiogenesis is an intermediary stage in wound repair [6], increased rates of cell proliferation would be expected to provide an acceleration of wound healing. A Phase II, double blind clinical study revealed that PsA methyl-ether treatment contributed to increased angiogenesis, granulation, and re-epithelialization above that of vehicle alone during early wound repair [7]. A secondary finding of the study revealed a suboptimal release of the drug into surrounding tissue and a potential reduction in efficacy due to non-specific binding.

Drug lipophilicity and formulation are critical components of biological drug assessments and there are many factors affecting the delivery of the drug to its site of action. These factors vary depending on the particular route of administration. For many routes, drugs need to be absorbed or transported to reach the site of action, which requires the crossing of one or more membranes and tissues [8]. A key feature that affects the drug transport across membranes is its solubility in an aqueous environment and in the lipid cell membrane, in addition to the drug’s ability to move from one phase to another (partition). Lipophilicity has been established to have a significant influence on drug potency [9] and it is suggested that a linear-free energy relationship should exist between a drug’s biological activity and lipophilicity [10].

The high lipophilicity and lack of aqueous solubility has hindered the study of the pseudopterosins in our laboratory for many years. The amphiphilic structure of PsA would be assumed to impart some aqueous solubility but in fact, the pseudopterosins have little to none and this limits the efficacy of the drug in biological model systems, as shown herein. Several methods have been explored to alter their physical properties (e.g., their solubility) and bioavailability in biological systems including the synthesis of pseudopterosin succinate salts and the production of alternative formulations. 

Cyclodextrins are a family of cyclic molecules composed of glucose monomers connected by α(1,4) glucosidic bonds. The toroidal molecular structure produces an interior cavity that is distinctly non-polar and an exterior that interacts favorably with aqueous environments making these compounds uniquely suited for lipophilic drug solubilization. Herein we describe a formulation of PsA in hydroxypropyl-β-cyclodextrin (HPβCD) (Figure 1C) that appears to structurally limit the presentation of the PsA molecule to its target. This significantly improves the potency *in vitro* and may be able to significantly improve the therapeutic index of these marine natural products in wound healing.

## 2. Results/Discussion

### 2.1. Log P as a Link to Specific Activity

Reverse phase HPLC (RP-HPLC) has been previously used to measure the logarithm of the octanol-water partition coefficient (log P) of many compounds [11]. To obtain the log P values recorded herein, the retention times of the compounds of interest (Table 1) along with the retention times of internal standards (toluene and triphenylene) were utilized. The resulting quantities were then substituted into Equation 1:

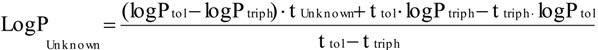
(1)
where the abbreviation tol refers to toluene, triph to triphenylene, and t to the retention time. Details concerning how this equation was derived have been described by Donovan and Pescatore [12].

**Table 1:** Structure and activity data for pseudopterosin analogs.

Analog number	Analog name	Direct log P	log(1/SA)
		(*n* = 4)	(*n* = 10)
**1**	Ps keto ketal [4]	3.95 ± 0.02	7.63 ± 0.06
**2**	arabinopyranoside of seco-PsA [13]	4.03 ± 0.05	7.69 ± 0.04
**3**	PsA methyl ether	4.37 ± 0.02	8.12 ± 0.02
**4**	PsA	4.42 ± 0.06	8.08 ± 0.01
**5**	iso-PsE	4.59 ± 0.05	8.02 ± 0.02
**6**	PsE	4.60 ± 0.06	7.88 ± 0.03

**Figure 2:**
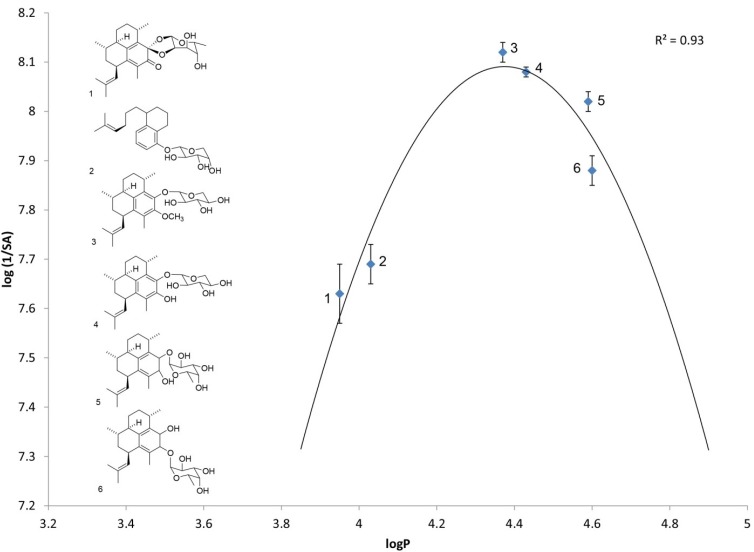
Parabolic relationship. Analysis of HPLC log P values and specific activity for pseudopterosin analogs **1**–**6** in the mouse ear edema model; data from Table 1.

The six pseudopterosin analogs shown in Table 1 and Figure 2, were chosen in anticipation that they would display a reasonably wide range of lipophilicities. As illustrated in Table 1, this proved to be the case. The log P values ranged from 4.60 ± 0.06 for pseudopterosin E (PsE) to 3.95 ± 0.02 for pseudopterosin keto ketal (Ps keto ketal, **1**). 

The compounds were then assessed using the standard phorbol myristate acetate (PMA) induced mouse ear edema assay [1,2] to determine their anti-inflammatory specific activity. In this assay, the specific activity of the drug, SA, is expressed as log(1/SA). For the mouse ear model, the molar concentration of the drug was calculated using Equation 2:

SA = (mol of drug) (∆ in edema from control)^−1^ (2)

where the difference in edema from control, ∆, refers to the difference in the average weight of the mouse ear between treated and untreated biopsies. The outcome, (1/SA), is expressed in units of milligrams of inhibited edema per mol of drug. Of the compounds tested, PsA methyl ether (structure **3**) registered the largest value for log(1/SA), a value of 8.12 ± 0.02 mg/mol, while pseudopterosin keto ketal (structure **1**), showed a significant decrease to a value of 7.63 ± 0.06, one of the lowest values measured [4,14]. Note Table 1. 

Compounds **1**–**6** were shown to display a parabolic response to the inhibition of mouse ear edema (Figure 2). From this graph, one can see that for the group of analogs tested, the log P value that results in the optimal activity is approximately 4.4. Thus, PsA methyl ether and PsA were found to have values that are near the optimum. This data represents the first suggestion that pseudopterosin lipophilicity may indeed be a limiting factor in the overall biological assessment since the structures with the largest and smallest log P values, PsE and Ps keto ketal **1** respectively, display the least biological activity. 

### 2.2. Traditional Formulation to Assess Cytotoxicity

Chronic wounds are characterized by a non-typical progression through the normal wound healing phases as a result of microbial colonization and other underlying pathologies. Increased proteolytic activity at the wound site caused by host inflammatory cells results in growth factor degradation and leads to slower healing rates [15,16,17]. In an effort to mimic this patho-physiological phenomenon we used conditions that were depleted of growth factors for all of the biological assays employed. One of the initial mechanisms involved in dermal wound healing is angiogenesis. For the *in vitro* studies described below, HUVEC cells [18,19] were utilized; they were selected primarily because of their angiogenic association with dermal wound healing and repair. HUVEC cells are very well characterized, thoroughly accepted, easily obtained, and reproducible cell types that can be used to demonstrate angiogenic effects. They are uniquely known for their increased sensitivity to solvent/vehicle toxicity, an attribute that is especially evident with the absence of growth factors. 

**Figure 3:**
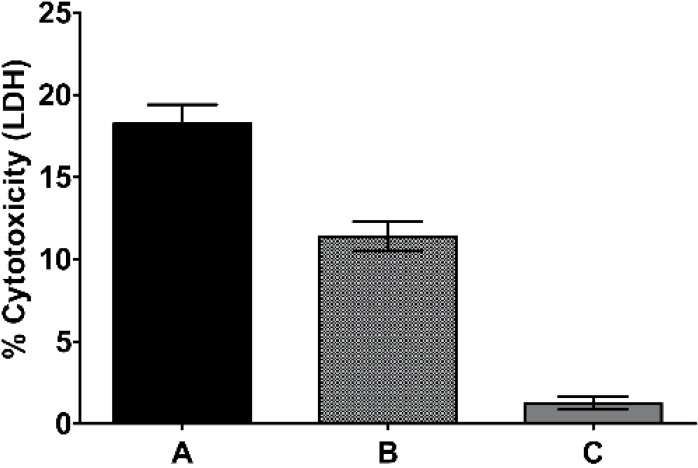
HUVEC cytotoxicity elicited by drug formulations. HUVEC LDH release with (**A**) 0.02% DMSO (**B**) 0.016% Cremophor EL and (**C**) 0.05% HPβCD after 42 h in growth factor depleted media at 37 °C. Data are presented as mean ± SEM (*n* = 4).

Cyclodextrins were introduced commercially for their unique ability to form inclusion complexes with non-polar, poorly soluble compounds and with an attendant increase in aqueous drug solubility and stability while imparting little-to-no toxicity [20,21]. In contrast, conventional vehicles used for the solubilization of drugs often produce cytotoxic effects in HUVEC cell cultures as shown by the release of a normally sequestered lactate dehydrogenase (LDH) (Figure 3). 

Dimethylsulfoxide (DMSO) is the most widely utilized solvent, and is used extensively in biological assays for attaining aqueous solubility for non-polar drug molecules. To determine the potential cytotoxic effects associated with DMSO, Cremophor EL, and HPβCD in a HUVEC system, we report three conditions that were chosen after considering the minimum required vehicle concentration for PsA (drug) solubility. Unexpectedly, DMSO (0.02%) and Cremophor EL^®^ (0.016%) alone induced substantial cytotoxicity, with values of 18.4% ± 1.6%, and 11.4% ± 0.9% of LDH release respectively, in growth factor depleted HUVEC cells (Figure 3). In contrast, we discovered that HUVEC cell cytotoxicity was insignificant with a formulation of HPβCD (0.05%) alone, thus indicating that HPβCD was strategically a good choice for use as a vehicle for pseudopterosin biological assessments.

### 2.3. Pseudopterosin Induces HUVEC Proliferation

Given that (a) PsA is readily available, (b) is a source of interest to researchers around the world, and (c) displays near optimal specific activity, we chose it for the studies presented herein. In HUVEC cellular assays, PsA has been shown to be cytotoxic at high concentrations in all formulation systems tested (>1.0 × 10^−5^ M) [1,22,23]. This cytotoxicity has been a limiting factor in efforts to expand the therapeutic index of the drug and occurred with all formulations tested in HUVEC cells at 1.5 × 10^−5^ M or above. It is unclear whether the cytotoxicity is due to non-specific binding of PsA or to a direct downstream effect related to the mechanism of action. 

**Figure 4:**
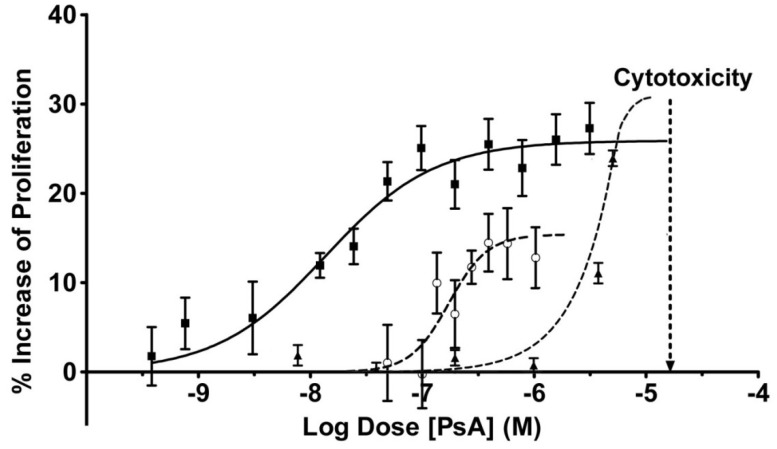
PsA Potently Activates HUVEC Proliferation. WST-1 assay of serum deprived HUVEC with various concentrations of PsA after 42 h in growth factor depleted media at 37 °C. ■ PsA: 0.05% HPβCD (solid line), ○ PsA: 0.016% Cremophor EL^®^ (discontinuous line), ▲ PsA: 0.02% in DMSO (dashed line). Note saturation kinetics. Cytotoxicity is illustrated as a vertical dashed line at 1.5 × 10^−5^ M in all vehicles tested. The percentage increase was calculated as [100 × (T/C)] where T/C is defined as Total/Control. Data are presented as mean ± SEM (*n* = 16).

We have discovered that various formulations of PsA elicit significantly different potency results in HUVEC proliferation assays (Figure 4). As illustrated in Figure 4, PsA in a 0.02% DMSO formulation displayed one of the least potent outcomes in HUVEC proliferation with an EC_50_ of approximately 3.1 × 10^−6^ M and a 30% ± 4.8% increase in proliferation after 42 h when compared to DMSO alone. This DMSO formulation of PsA resulted in a narrow therapeutic index, defined as the range between the EC_50_ of the proliferative effect and the cytotoxic effects of the drug. The confined therapeutic index resulted in the inability to decouple the beneficial effects of the drug from the cytotoxic effects in DMSO. A 0.016% Cremophor EL formulation of PsA produced an EC_50_ of 1.728 × 10^−7^ M, nH = 2.51, but afforded a substantially weaker physiological response in the HUVEC model (Figure 4; increased HUVEC proliferation by 15%). Formulation into Cremophor EL resulted in improved cell proliferation potency, but continued to closely couple the cell proliferation effects with cytotoxicity. This was indicated by the lack of a sustained maximal effect before the onset of cytotoxicity. 

*In contrast to the other formulations*, a 0.05% HPβCD formulation of PsA produced significant increases in potency and an overall therapeutic index that was *substantially improved over that of each of the other formulations tested*. Analysis of the data showed an EC_50_ of 1.34 × 10^−8^ M with *K*_d_ = 1.45 × 10^−8^ M, nH = 0.95 and an approximately 25% ± 3.4% maximal increase in HUVEC proliferation. The increased drug potency was also accompanied by an increase in overall percentage of cell proliferation compared to other formulations, one that was sustained over two orders of magnitude before cytotoxicity is encountered. *Thus we see that for the first time, a substantial uncoupling of cell proliferation and cytotoxicity is observed when PsA is formulated in HPβCD*. In addition, this formulation provides the first evidence of a receptor mediated effect due to the saturation effect observed between 10^−7^ and 10^−5^ M (Figure 4), as well as the pseudo-first order kinetics observed throughout the dose response in the absence of cytotoxicity. 

**Figure 5:**
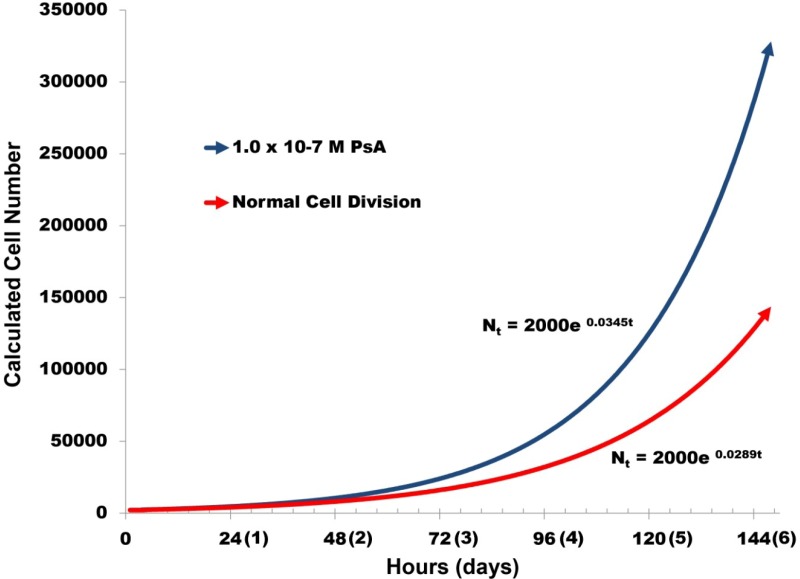
Estimated Impact of PsA Treatment on Cell Proliferation. Cell division estimated from proliferation assay data (note Figure 4) at 1.0 × 10^−7^ M PsA (25% increase) and normal cell division (control value). The supplier, Lonza Ltd. indicates that the doubling rate for HUVEC cells is 24 h.

At first glance the increase (25% ± 3.4%) in maximal HUVEC proliferation over 42 h appears to be relatively insignificant. In an attempt to provide a more tangible assessment for the increase, we estimated cell division by using the equation:

*N_t_* = *N*_initial_ e^(rate × time)^ (3)

where *N_t_* refers to the estimated cell count at time *t*, *N*_initial_ is the initial number of cells, and rate refers to the rate of cell division per hour. The known doubling time for HUVECs provided by the supplier is 24 h. Using this information we calculated both the rate for the control (normal cell division) and PsA treated sample to provide cell division estimate curves (Figure 5). In this manner we estimate that after 5 days, cells treated with 1.0 × 10^−7^ M PsA ought to reach 125,000, a value that is more than double that of the control. 

The proposed increase in receptor delivery efficiency is in accord with the increase in biological potency observed in the HUVEC proliferation assays. One possible orientation of the drug is illustrated (Figure 1C). Here, the hydrophobic tricyclic diterpene core resides on the interior of the cyclodextrin core while the sugar unit is directed toward the outside where it may engage in hydrogen bond interactions with the hydroxypropyl appendages of the cyclodextrin molecule.

### 2.4. PsA Inhibits Normal HUVEC Migration

The *in vitro* scratch assay is commonly used to simulate cellular events that accompany the presence of a wound [24]. Here, a thin scratch made in a cellular monolayer mimics the wound. The migration of cells in response to the scratch is then monitored using microscopic images at various time points to determine the rate of migration. 

**Figure 6:**
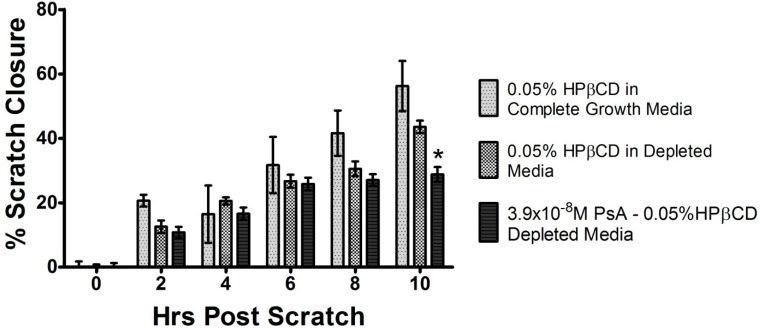
PsA inhibits HUVEC Cell Migration. Quantitative scratch assay of HUVEC migration from microscopic images taken at 400× magnification. Serum deprived HUVEC with 0.05% HPβCD in complete growth media (light grey); 0.05% HPβCD in depleted media (dark grey); 3.9 × 10^−8^ M PsA in 0.05% HPβCD (black) over 10 h at 37 °C. * Indicates significance of *p* value <0.01. Data are presented as mean ± SEM (*n* = 16). Note that it is impossible to perform these experiments using PsA alone *i.e.*, without DMSO or Cremophor, or the cyclodextrin being present, due to its low solubility in an aqueous medium.

As shown in Figure 6, the migration of HUVEC cells is significantly impaired in response to treatment with PsA in 0.05% HPβCD (note the black vertical bar). In 0.05% HPβCD and under full growth factor conditions, the migration rate of HUVEC cells correspond to 16.69 ± 0.98 µm h^−1^ in response to a monolayer scratch (light grey bar in Figure 6). The removal of growth factors from the media does not lead to a significant reduction in the migration rate compared to full growth factor conditions (15.01 ± 1.19 µm h^−1^ *vs.* 16.69 ± 0.98). In contrast, the administration of a low concentration of PsA (3.9 × 10^−8^ M) in 0.05% HPβCD results in significant decreases in the rate of HUVEC cell migration with a rate of 9.98 ± 1.2 µm h^−1^ (a 27.5% and 14.7% decrease in migration from complete growth media and depleted media respectively, at 10 h) and which slow significantly after 6 h. The difference (*p* < 0.01) from the growth factor depleted control is most significant at 10 h, an observation that may indicate a lagging molecular mechanism at work. We hypothesize that cell migration is balanced by cell proliferation and that the two processes do not occur simultaneously. If this is so, then the change in cell migration may be due to increased rates of proliferation that diminish cell migration. Studies designed to gain a detailed mechanistic understanding of these observations are underway and the results will be reported in due course.

## 3. Materials and Methods

### 3.1. Cell Culture and Reagents

*P. elisabethae* was generously donated by Professor William Fenical from a collection obtained from Sweetings Cay in the Bahamas. Pooled human umbilical vein endothelial cell (HUVEC) cultures, EGM-2 endothelial cell growth media bullet kit, 0.25% trypsin-EDTA solution, and all tissue culture supplies were purchased from Lonza Laboratories (Walkerville, MD, USA). Tissue culture treated flasks and 96-well plates were purchased from Greiner Bio-One (Monroe, NC, USA). Hydroxypropyl-beta-cyclodextrin was purchased from Acros Organics Co. Pre-mixed WST-1 reagent was purchased from Clontech Laboratories Inc. (Mountain View, CA, USA). All other chemicals used were of tissue culture or best grade available.

### 3.2. Equipment

HPLC recordings were achieved using a Hewlett Packard HP/Agilent 1100 HPLC system connected to a 1 cm × 4.6 mm, 5 µm aPHera™ C18 Polymer HPLC column (catalog number 56130AST). Colorimetric assays were performed on a Molecular Devices *V*_max_ microplate reader. 

### 3.3. Extraction and Purification from *P. elisabethae*

Samples of air-dried *P. elisabethae* were further dried under reduced pressure and desiccation for several months to achieve maximum dryness. The resulting samples were ground to a fine powder and extracted with 1:1 CH_3_OH:CH_2_Cl_2_ followed by filtration to obtain a crude pseudopterosin mixture consisting of Ps A, B, C, and D. Base hydrolysis of the mixture was then performed using the method described by Mydlarz *et al.* [25] to yield semi-pure PsA. This material was then purified by reverse phase HPLC using an acetonitrile/H_2_O gradient (60:40 to 100% acetonitrile over 25 min) with UV detection at 281 nm.

### 3.4. Biological Activity-Inhibition of Inflammation

Male *Swiss Webster albino* mice were briefly anesthetized with halothane. Compounds were topically applied to the inside pinnae of the ears of the mice in a solution containing the edema-causing irritant PMA. PMA alone (2 µg/ear) or in combination with the test compound (50 µg/ear) were dissolved in acetone and applied to the left ears (5 mice per treatment group, *n* = 10/compound), and acetone alone was applied to all right ears [26]. After 3 h and 20 min incubation, the mice were euthanized, the ears removed and a 6 mm diameter biopsy was taken from the center of each ear and immediately weighed. Edema was measured by subtracting the weight of the right ear (acetone control) from the weight of the left ear (treated). Compounds that were not soluble in 100% acetone were dissolved in 20% ethanol: 80% acetone v/v. Results were recorded as % decrease (inhibition) in edema relative to the PMA control group edema. (Our previous studies have concluded that PsA does not block PMA when it is administered concurrently; the present studies followed previously published investigations [1,2].)

### 3.5. Formation of Cyclodextrin Inclusion Complex

Hydroxypropyl-beta-cyclodextrin (HPβCD) was added to DI-water at a concentration of 20% w/v. The mixture was then vortexed and sonicated briefly to yield a colorless, transparent, slightly viscous liquid. Separately, a given quantity of pseudopterosin A (PsA) was then solubilized in 100% methanol to yield a yellow, transparent liquid. An aliquot of this solution was then added to a culture tube and dried under inert atmosphere yielding an off-white solid. The previously prepared hydroxypropyl-beta-cyclodextrin solution was then added and vortexed for 10 min. The mixture was orbitally shaken at 4 °C for at least 7 days prior to use to ensure equilibration of the cyclodextrin drug complex. The final product at 0.05% HPβCD was a clear solution that was found to be soluble in saline or tissue culture medium. Stability was conserved at least 14 days, as observed by HPLC and NMR.

### 3.6. Drug Solubilization Verification

Following the formulation of PsA into DMSO, Cremophor EL, or HPβCD, samples were prepared at their final concentration in EBM-2 basal media to determine the similarity of drug solubility between different formulations. Reverse phase HPLC was utilized as described in Section 3.3 above to confirm the quantity of solubilized drug that remained unchanged between formulations. (Note: It is impossible to perform these experiments using PsA alone due to its low solubility in an aqueous medium.) 

### 3.7. HUVEC Cell Culture

All cell culture experiments were performed using HUVEC cells initially seeded at 3 × 10^5^ cells/75 cm^2^ flask and fed every 1–2 days with fresh EGM-2 complete culture media (EBM-2 basal media with hVEGF, hFGF, hIGF, hEGF, hydrocortisone, ascorbic acid, heparin, 2% FBS, and GA-1000). For each experiment cells were split at 80% confluence. All experiments were conducted between generations 4 and 10. Starvation media consisted of EBM-2 basal media with 0.1% FBS, and GA-1000 (antibiotic mixture). Growth factor depleted media consisted of EBM-2 basal media with 2% FBS, hydrocortisone, ascorbic acid, heparin, and GA-1000.

### 3.8. WST-1 Cell Proliferation Assay

HUVEC were harvested from a T-75 plate by trypsinization and resuspended in EGM-2 complete culture media. Cells were plated at 500 cells per well of a 96-well plate and incubated overnight at 37 °C in a 5% CO_2_ atmosphere. EGM-2 complete culture media was removed and replaced with starvation media for 8 h. During this time, drug dilutions were prepared in growth factor depleted media. Starvation media was replaced with drug dilution in growth factor depleted media and incubated at 37 °C in a 5% CO_2_ atmosphere. After 41 h, 10 µL of WST-1 reagent was added to each well and incubated for 1 h. Plates were then read on a multiplate reader at 450 nm with background reference of 650 nm.

### 3.9. Scratch Migration Assay

HUVEC were harvested from a T-75 plate by trypsinization and resuspended in EGM-2 complete culture media and were plated into T-25 cell culture flasks and allowed to grow to approx 90% confluence. EGM-2 complete culture media was then removed and replaced with starvation media for 8 h. A uniform scratch in the monolayer was then produced using a sterile pipette tip approximately 500 µm in width. Starvation media was replaced with 5 mL of either PsA in drug dosing media, or HPβCD in drug dosing media, or HPβCD in EGM-2 complete culture media. Microscopic images were taken at 400 times magnification every 5 min beginning at *t* = 0, and continuing until *t* = 10 h. Images were analyzed by determining the distance between cells on either side of scratch over time. Percent scratch closure was set to be a function of 1 − (average distance at each time point/average width of scratch). 

### 3.10. Statistical Analysis

Graphs and statistical analysis performed using Graphpad Prism ver. 5.0. A simple student’s t-test was performed because there is one measurement-variable namely, percent closure, and one nominal variable, viz., the presence or absence of PsA (note Figure 6). The null hypothesis (*i.e.*, that there is *no* *difference* between the two groups) is that the mean percent closure for the two treatments is the same. 

## 4. Conclusion

In the present study we sought to provide evidence showing that pseudopterosins in fact do show a direct relationship between lipophilicity and specific activity in an *in vivo* model. This objective led us to pursue alternative formulations which show that PsA adminstration in HPβCD potently activates proliferative effects in HUVECs. If one defines bioavailability to be the degree to which a drug or other substance becomes available to a target after administration [27], we believe that we have met this threshold in our demonstration of an increased therapeutic index. Our results provide evidence to suggest that PsA incorporation into a HPβCD complex may uniquely create a constrained PsA molecule that results in increased potency and selectivity. This has allowed for the very first decoupling of the cell proliferation and cytotoxic effects of the drug, and has afforded further elucidation of PsA’s mechanism of action. We believe that the benefits observed by HPβCD in these experiments transcend mere increases in drug solubility and may be unique to pseudopterosins. Furthermore, we anticipate that PsA formulation into HPβCD would have an *in vivo* impact specifically by minimizing the non-specific binding of the drug, which currently limits its therapeutic index in a clinical setting. Topical formulation of this cyclodextrin formulation into a crème or aqueous hydrogel would be expected to have greater efficacy in *in vivo* applications than a conventional formulation. 

We believe these data illustrate that pseudopterosins, when solubilized in cyclodextrins are potentially promising drug candidates for the treatment of a variety of chronic conditions [28,29]. In addition preparation of PsA and other amphiphilic compounds in cyclodextrin solutions may allow for new studies of these compounds in a variety of contexts that were previously restricted because of poor water solubility.
